# Evaluation of real-world mobility in age-related macular degeneration

**DOI:** 10.1186/1471-2415-15-9

**Published:** 2015-01-30

**Authors:** Sabyasachi Sengupta, Angeline M Nguyen, Suzanne W van Landingham, Sharon D Solomon, Diana V Do, Luigi Ferrucci, David S Friedman, Pradeep Y Ramulu

**Affiliations:** Wilmer Eye Institute, Johns Hopkins University, 600 North Wolfe St, Maumenee B-110, Baltimore, MD 21287 USA; Truhlsen Eye Institute, University of Nebraska Medical Center, Omaha, NE USA; The Longitudinal Studies Section, Clinical Research Branch, National Institute on Aging, National Institutes of Health, Bethesda, MD USA; Johns Hopkins Bloomberg School of Public Health, Baltimore, MD USA; Dana Center for Preventive Ophthalmology, Johns Hopkins University, Baltimore, MD USA

**Keywords:** Age-related macular degeneration, Physical activity, Mobility

## Abstract

**Background:**

Previous research has suggested an association between poor vision and decreased mobility, including restricted levels of physical activity and travel away from home. We sought to determine the impact of age-related macular degeneration (AMD) on these measures of mobility.

**Methods:**

Fifty-seven AMD patients with bilateral, or severe unilateral, visual impairment were compared to 59 controls with normal vision. All study subjects were between the ages of 60 and 80. Subjects wore accelerometers and cellular network-based tracking devices over 7 days of normal activity. Number of steps taken, time spent in moderate-to-vigorous physical activity (MVPA), number of excursions from home, and time spent away from home were the primary outcome measures.

**Results:**

In multivariate negative binomial regression models adjusted for age, gender, race, comorbidities, and education, AMD participants took fewer steps than controls (18% fewer steps per day, p = 0.01) and spent significantly less time in MVPA (35% fewer minutes, p < 0.001). In multivariate logistic regression models adjusting for age, sex, race, cognition, comorbidities, and grip strength, AMD subjects showed an increased likelihood of not leaving their home on a given day (odds ratio = 1.36, p = 0.04), but did not show a significant difference in the magnitude of time spent away from home (9% fewer minutes, p = 0.11).

**Conclusion:**

AMD patients with poorer vision engage in significantly less physical activity and take fewer excursions away from the home. Further studies identifying the factors mediating the relationship between vision loss and mobility are needed to better understand how to improve mobility among AMD patients.

## Background

Regular physical activity and travel outside one’s home are strongly associated with psychological well-being and physical health [1]. Prior research has shown that individuals with mobility restrictions experience a lower quality of life as a result of decreased personal autonomy and independent living and have higher morbidity and mortality, especially among the elderly [1–4]. Identifying high-risk populations in which these mobility measures are compromised is critical so that they can be targeted for interventions to safely increase their mobility and ultimately improve their health and sense of well-being.

Previous research utilizing accelerometers to objectively measure physical activity has demonstrated that impairment of both central and peripheral vision are associated with significantly decreased physical activity [5–7]. Additionally, studies analyzing responses to life space questionnaires have concluded that patients with vision impairment from diseases affecting central and peripheral vision are likely to restrict the frequency of entering different life spaces (i.e. bedroom, driveway, neighborhood, town, out of town, etc.) [8]. Other studies have focused more specifically on age-related macular degeneration (AMD) as a risk factor for decreased mobility, as it is a leading cause of central vision loss in older adults and occurs independently of systemic comorbidities that could also lead to mobility difficulty. These studies have demonstrated that AMD is associated with worse balance, more frequent falls, and greater restriction of driving [9–11], but have not investigated whether AMD patients are less physically active or restrict how often they leave home. While such studies have evaluated isolated risk factors for mobility-related adverse outcomes, studying life space offers unique information about the extent of one’s travel within the environment. Our use of accelerometers and cellular tracking annuls the reliance upon recall through questionnaires and provides accurate real-world quantitative measurements of travel and physical activity which are less subject to recall bias.

Real-world, objective measurements of physical activity and travel outside the home provide unique insights into health outcomes not provided by questionnaires. For example, previous research has demonstrated that physical activity as measured by an accelerometer is more correlated with body-mass index (BMI), blood pressure, triglyceride levels, and waist circumferences than self-reported physical activity, thus demonstrating that accelerometer-defined physical activity is more reflective of true physical activity levels [10]. Such findings argue for the fact that travel from the home should also be measured objectively whenever possible. Here, we compared a group of AMD patients with at least some vision impairment to control subjects without vision impairment using (1) accelerometers to quantify physical activity in terms daily steps and time spent in moderate-to-vigorous activity (MVPA), and (2) cellular network-based tracking devices to quantify travel from the home in terms of the likelihood of leaving one’s home on a given day and time spent outside the home.

## Methods

The study was approved by the Johns Hopkins Medical Institutions’ Institutional Review Board, and written informed consent was obtained from all study subjects. Subjects were recruited and completed study procedures between July 2009 and June 2012.

### Study participants

All patients meeting the enrollment criteria were approached in retina and glaucoma clinics at the Wilmer Eye Institute at Johns Hopkins Hospital for possible study participation on days when a research coordinator was available for recruitment. Patients refusing to participate for reasons related to difficulty transporting to the hospital were assured that they would be provided help with transportation free of charge. Eligible patients were between the ages of 60 and 80 years. AMD subjects had a diagnosis of bilateral AMD with evidence of either geographic atrophy or choroidal neovascularization in at least one eye and with drusen and/or retinal pigment atrophy in both eyes. Visual acuity (VA) was required to be 20/32 or worse in both eyes, or worse than 20/200 in one eye regardless of the second-eye vision. Control subjects had a diagnosis of glaucoma suspect or ocular hypertension and had a presenting VA of better than 20/40 in both eyes, a mean deviation (MD) better than −5 decibels (dB) in both eyes using the Swedish interactive thresholding algorithm (SITA) standard Humphrey 24–2 VF test, and a glaucoma hemifield test (GHT) result other than “Outside Normal Limits” in both eyes. Previous work has shown this group to be highly similar with regards to visual parameters when compared to elderly subjects analyzed as part of population-based studies [12].

### Evaluation of physical activity

Physical activity was evaluated over 7 days of normal activity using an omnidirectional accelerometer (Actical; Respironics, Inc, Andover, MA). The methods used during the study have been described in detail elsewhere [7]. In summary, subjects were instructed to clip an accelerometer to their waistband in front of their hip during all waking hours except when swimming or bathing. Motion detected by the accelerometer was used to record steps, and to summarize data as counts reflecting total detected acceleration in arbitrary units. The physical activity level occurring over each study minute was classified as sedentary, light, moderate, or vigorous using cut points previously defined by Colley and Tremblay [13]. A value of 1,535 counts/minute or more was classified as MVPA.

### Evaluation of travel away from home

Travel habits were assessed over the same 7-day period as the accelerometer assessment using a cellular network-based tracking device (pTrac Pro, Brickhouse Security, New York, NY). Subjects were instructed to clip the tracking device to their waistband during waking hours. The tracking device was set to record the subject’s longitude and latitude every 15 minutes between 7 am and 11 pm. Each provided location was defined as “home” if the location was within one-third of a mile (the minimum device resolution) of the patient’s given home address, and “away” if the location was at least one-third of a mile from the home location. Home/away designations were then used to quantify both excursions and time away from home. Details regarding the function and validity of the tracking device, and a more detailed explanation regarding how excursions and time spent away from home were determined, are provided elsewhere [7, 14].

### Ensuring and evaluating device compliance

Subjects were called each morning on each of the 7 study days to maximize compliance with device wear. Compliance was also assessed by estimating accelerometer wear time, taken as the interval between the first and last minutes showing non-zero accelerometer counts for the given day. Accelerometer data were excluded from study days in which fewer than 8 hours of estimated wear time were measured. Cellular tracking data were excluded if the time from the first to last provided location was less than 12.8 hours (indicating poor device or battery function) or if accelerometer data were excluded for the same study participant (suggesting that both devices were not worn). Accelerometer and cellular tracking data were also excluded from study days in which subjects admitted non-compliance during their daily reminder phone calls.

### Measurement of vision and covariates

Monocular presenting VA was measured using the Early Treatment Diabetic Retinopathy Study (ETDRS) chart with standardized illumination, and better-eye VA was converted to the logarithm of the minimum angle of resolution (logMAR) for use in statistical analysis [15]. Binocular contrast sensitivity (CS) was measured using the Pelli-Robson chart at 1 meter with subjects wearing their usual correction, and was converted into log units (log CS) for analysis [16]. Both eyes were examined after pupillary dilation to assess for lenticular changes in phakic eyes, or posterior capsular opacification (PCO) in pseudophakic eyes, as previously described [17, 18].

Demographic information including age, gender, race, employment status, and years of education completed were collected by self-report. Cognitive ability was assessed using the Mini Mental Status Exam (MMSE) for the Visually Impaired (scored from 1–22) [19]. Depressive symptoms were detected using the Geriatric Depression Scale Short Form, and subjects demonstrating 6 or more positive responses were considered to have depressive symptoms [20]. Medical comorbidities were assessed using a standardized structured medical history questionnaire and summarized as the number of comorbid conditions present [21]. Queried comorbidities included arthritis, broken or fractured hip, back problems, heart attack/myocardial infarction, angina/chest pain, congestive heart failure, peripheral vascular disease, hypertension, diabetes, emphysema, asthma, stroke, Parkinson’s disease, cancer (other than skin cancer), and vertigo/Meniere’s. Since driving is a primary means to leave the home, driving habits were evaluated with a questionnaire from the Salisbury Eye Evaluation Driving Study (SEEDS) [22]. Specifically, subjects were asked, “Have you driven a car in the past three months?” to assess driving cessation.

Weather information, including daily temperatures and rainfall, was gathered through the North East Regional Climate Center (Cornell University, NY) and summarized as the percentage of study days with 0.2 or more inches of rainfall and the percentage of study days with an average daily temperature below 45°F (representing the 33rd percentile for average daily temperature in the study). Weather information for each subject was inferred based upon the weather station closest to the home location.

### Statistical analysis

Sample size was set at 60 subjects per group to allow for the detection of 25% less physical activity and 25% fewer excursions in the AMD group as compared to the control group with 80% power. Calculations for sample size were made based upon knowledge of previously reported levels of physical activity amongst older adults and upon the assumption that 7 ± 3 excursions occurred per week in the control group [23]. Group differences for continuous variables were evaluated using the Student’s t-test or the Wilcoxon rank-sum test. Chi-square analysis was used to assess differences in categorical variables.

Analyses of (1) steps taken per day, (2) minutes spent in MVPA per day, and (3) minutes spent outside of the home per day were performed using univariate and multivariate negative binomial regression models. Odds of not leaving the home were analyzed with univariate and multivariate logistic regression models. In all models, each person-day was considered as a separate observation, and generalized estimating equations (GEE) were used to account for correlation between activity levels across different days from the same individual [7, 24]. GEEs are an extension of generalized linear models which account for within-subject covariance in order to model the mean response of each participant [25]. Using GEE regression accounts for the fact that there may be correlated physical activity and excursion data between the 7 days tested for the same participant. Outcomes of negative binomial models are expressed as rate ratios (RR) while logistic regression model outcomes are expressed as odds ratios (OR). Covariates were included in multivariate models if there was prior evidence linking the variable to activity levels (age, race, gender) [7] or if a significant association was noted in univariate analyses (p < 0.05).

Potential covariates which might mediate the relationship between vision and activity restriction (i.e. driving status, depressive symptoms, employment status), or which might occur as a result of decreased activity (i.e. depressive symptoms, BMI), were not included in multivariate models. For instance, driving may mediate the relationship between vision loss and mobility, given that driving is a primary method for leaving the home and that rates of physical activity are significantly higher outside the home than inside the home [14]. While the presence of depressive symptoms in individuals with eye disease may lead to decreased physical activity [26], depression itself has been found to also be a consequence of physical activity restriction [27]. A similar argument can be made about employment status and physical activity [28]. Therefore, the metrics for evaluating depressive symptoms, employment status, and driving status were not deemed appropriate to be added to our models seeking the association between vision and physical activity. It is likely that the threshold for cataract removal increased with severity of AMD given that cataracts in eyes with advanced disease may not improve vision. In this case, cataract would serve as a marker for more advanced AMD and would not reflect behavioral changes associated with the cataract itself. Therefore, the association of cataract with mobility outcomes was explored in univariate analyses, but cataract status was not included as a covariate in our primary multivariate regressions models. Analyses were performed using STATA 13.0 (StataCorp, College Station, TX).

## Results

Fifty seven AMD subjects and 59 control subjects were included in the study on the basis of having at least one valid study day of accelerometer and cellular tracking data. Subjects in both groups had a comparable number of days with usable cellular tracking data (6.2 ± 0.2 + 1.6 in controls vs. 5.7 ± 0.2 in AMD subjects, p = 0.10) while control subjects had a greater average number of days with usable accelerometer data as compared to AMD subjects (6.7 ± 0.1 in controls vs. 6.2 ± 0.2 in AMD subjects, p = 0.02). Subjects with AMD were significantly older, had worse VA and CS, and were more likely to be white when compared to control subjects (p ≤0.01 for all) (Table 1). All other demographic and health-related variables were similar across AMD status.

**Table 1:** **Characteristics of study participants by disease status**

	Controls	AMD	***P***value
	(n = 59)	(n = 57)	
Vision*			
Better eye VA, logMAR	0.08 (0.12)	0.43 (0.32)	**<0.001**
Worse eye VA, logMAR	0.18 (0.14)	1.05 (0.46)	**<0.001**
Binocular CS, log units	1.88 (0.01)	1.46 (0.02)	**<0.001**
Sig. cataract/PCO, either eye, %	21.8	32.1	0.23
Sig. cataract/PCO, both eyes, %	10.9	15.1	0.52
Demographics*			
Age, years	69.3 (5.3)	74.4 (5.2)	**<0.001**
Female gender, %	62.7	57.9	0.60
White race, %	74.6	91.2	**0.01**
Education, years	15.5 (2.2)	15.0 (1.9)	0.13
Unemployment, %	61.0	75.4	0.10
Health/cognition/driving*			
MMSE-VI score	20.8 (1.4)	20.6 (1.7)	0.76
Comorbid illnesses (#)	2.49 (1.6)	2.50 (1.6)	0.92
Depressive symptoms, %	5.1	5.3	0.97
Driving help, %	77.8	73.7	0.59
Grip strength, kg	27.2 (9.3)	28.2 (9.3)	0.57
**Weather**			
Temperature <45°F, % days	3.23 (4.2)	2.50 (4.0)	0.45
Rainfall >0.2 mm, % days	1.73 (1.81)	1.75 (1.74)	0.96

AMD = age-related macular degeneration; VA = visual acuity; CS = contrast sensitivity; logMAR = logarithm of the minimum angle of resolution; Sig. = significant; PCO = Posterior Capsular Opacification; MMSE-VI = Mini-mental status examination for the visually impaired; °F = degrees Fahrenheit; mm = millimeters.

*Continuous variables reported as mean (SD). Values in bold indicate p < 0.05.

**Figure 1:**
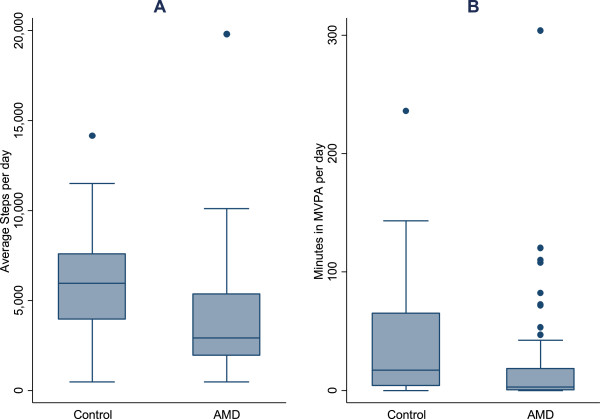
**Average number of steps per day and time spent in moderate-to-vigorous physical activity (MVPA) per day by AMD status. (A)** Average number of steps per day and **(B)** minutes spent in MVPA per day by AMD status.

With regards to accelerometer outcomes, individuals with AMD walked less (median = 2,930 steps per day, interquartile range [IQR] = 1,938 – 5,374) than controls (median = 5,960 steps per day, IQR = 3,956 – 7,601) (p < 0.001) and spent less time engaged in MVPA (median = 3.0 minutes/day, IQR = 1 – 18.5 minutes) than controls (median = 17.1 minutes/day, IQR = 4 – 65 minutes) (p < 0.001) (Figure 1). Multivariate GEE models adjusting for age, gender, race, comorbidities and education showed that AMD patients walked 18% less than controls (95% CI = 4 – 31%, p = 0.02; Table 2). In separate multivariate models, fewer daily steps was associated with worse better-eye VA (5% reduction in daily steps per 0.1 logMAR increment, 95% CI = 1 – 10%, p = 0.05) and with worse CS, though not at a statistically significant level (4% reduction in daily steps per 0.1 logCS decrement, 95% CI = 0 – 9%, p = 0.09). Non-visual factors associated with decreased physical activity included lower educational attainment and the presence of comorbid illness (Table 2).

**Table 2:** **Association of AMD and visual metrics with daily steps walked, univariate and multivariate analyses**

Variable	Interval	Univariate analysis		Multivariate analysis	
		RR	***P***value	RR*	***P***value
**Vision**					
AMD	Present	0.67	**0.007**	0.82	**0.02**
Better eye VA	0.1 logMAR worse	0.93	**0.004**	0.95	**0.05**
Binocular CS	0.1 log decrement	0.95	**0.014**	0.96	0.09
Sig. cataract/PCO	Present	0.83	0.29	---	---
**Demographics**					
Age	10 years older	0.81	0.11	1.03	0.84
Gender	Female vs. male	0.96	0.81	0.99	0.96
Race	White vs. non-White	1.02	0.92	1.10	0.64
Education	4 years more	1.35	**0.02**	1.30	**0.04**
Employed	Yes vs. No	1.28	0.14	---	---
**Health/cognition**					
Comorbidities	1 or more illness	0.87	**<0.01**	0.89	**<0.01**
Depressive symptoms	Present vs. absent	0.45	**0.02**	§§	§§
BMI	1 unit higher	0.97	**0.01**	‡‡	‡‡
MMSE – VI score	5 points worse	0.81	0.37	---	---
**Weather**					
Temperature <45°F	Yes vs. No	0.11	0.54	---	---
Rainfall >0.2 mm	Yes vs. No	0.02	0.6	---	---

RR = Rate ratios; AMD = age-related macular degeneration; VA = visual acuity; CS = contrast sensitivity; logMAR = logarithm of the minimum angle of resolution; PCO = Posterior Capsular Opacification (either eye); BMI = body mass index; MMSE-VI = Mini-mental status examination for the visually impaired; °F = degrees Fahrenheit; mm = millimeters.

*RR for vision variables were each derived from separate multivariate models including all non-visual covariates shown. RR for non-visual variables were derived from the multivariate model containing AMD as the only vision variable. Values in bold indicate p < 0.05.

§§ - Employment and depression were not included in the multivariate models as these may occur as a result of AMD and do not directly influence effect of vision related parameters on physical activity.

‡‡ BMI was not included in the models as it may be a cause or result of reduced physical activity and does not directly influence the association between vision-related parameters and physical activity.

Additional multivariate models adjusting for age, gender, race, comorbid illness and education were run to investigate the association between AMD and time engaged in MVPA. AMD subjects spent 35% less time in MVPA as compared to controls (95% CI = −24 to −44%, p < 0.001; Table 3). In separate multivariate models, subjects with worse VA engaged in less daily MVPA (11% less MVPA per 0.1 logMAR increment, 95% CI = −7 to −16%, p < 0.001), as did subjects with worse CS (13% less MVPA per 0.1 log CS decrement, 95% CI = −9 to −18%, p < 0.001). White race and higher educational attainment were also significantly associated with more time spent in MVPA, while female gender and presence of comorbidities were significantly associated with lower amounts of MVPA. Additional multivariate models were run to examine whether AMD status and vision remained associated with less MVPA when cataract status was added to the model. When cataract status was added to multivariate models, AMD status, VA, and CS remained associated with daily MVPA (p < 0.05 for all), while the presence of cataract/PCO was also associated with less MVPA (44% less MVPA when present, 95% CI = 22% - 60%, p = 0.001 in multivariate models containing cataract/PCO, AMD, and other non-visual predictors as independent variables).

**Table 3:** **Effect of AMD and vision metrics on time spent in moderate-to-vigorous physical activity (MVPA) per day**

Variable	Interval	Univariate analysis		Multivariate analysis	
		RR	***P***value	RR*	***P***value
**Vision**					
AMD	Present	0.52	**<0.001**	0.65	**<0.001**
Better eye VA	0.1 logMAR worse	0.87	**<0.001**	0.89	**<0.001**
Binocular CS	0.1 log decrement	0.85	**<0.001**	0.87	**<0.001**
Sig. cataract/PCO	Present	0.58	**0.001**	§§	§§
**Demographics**					
Age	10 years older	0.63	**<0.001**	1.00	0.98
Gender	Female vs. male	0.71	**0.02**	0.62	**<0.01**
Race	White vs. non-White	1.63	**0.01**	2.01	**<0.001**
Education	4 years more	2.22	**<0.001**	2.33	**<0.001**
Employed	Yes vs. No	1.94	**<0.001**	§§	§§
**Health/cognition**					
Comorbidities	1 or more illness	0.73	**<0.001**	0.73	**<0.001**
Depressive symptoms	Present vs. absent	0.08	**<0.001**	§§	§§
BMI	1 unit higher	0.90	**<0.001**	‡‡	‡‡
MMSE – VI score	5 points lower	0.96	0.87	---	---
**Weather**					
Temperature <45°F	Yes vs. No	0.04	0.93	---	---
Rainfall > 0.2 mm	Yes vs. No	0.07	0.57	---	---

RR = Rate ratios; AMD = age-related macular degeneration; VA = visual acuity; CS = contrast sensitivity; logMAR = logarithm of the minimum angle of resolution; PCO = Posterior Capsular Opacification; BMI = body mass index; MMSE-VI = Mini-mental status examination for the visually impaired; °F = degrees Fahrenheit; mm = millimeters.

*RR for vision variables were each derived from separate multivariate models including all non-visual covariates shown. RR for non-visual variables were derived from the multivariate model containing AMD as the vision variable. Values in bold indicate p < 0.05.

§§ - Employment, depression, and cataract/PCO were not included in the multivariable models as these occur as a result of AMD and do not directly influence effect of vision related parameters on physical activity.

**Figure 2:**
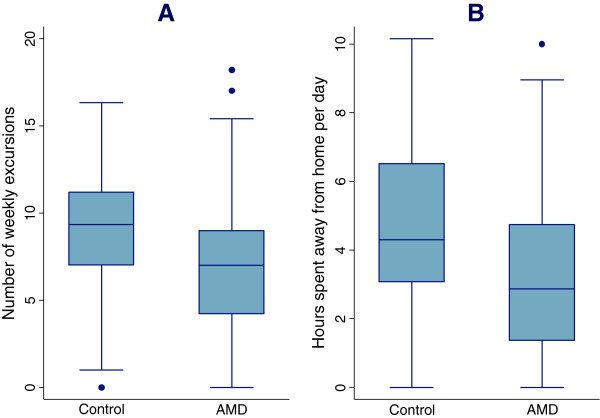
**Excursions made per week and hours spent away from home per day by AMD status. (A)** Excursions made per week and **(B)** hours spent away from home per day by AMD status.

With regards to travel outside the home, individuals with AMD undertook significantly fewer excursions over the study week (5.7 ± 3.7 excursions, range = 0 – 17) as compared to controls (8.2 ± 3.7 excursions, range = 0 – 16) (p < 0.001) (Figure 2). AMD subjects also spent fewer daily hours away from home (3.4 ± 3.3 hours, range = 0 – 14.4 hours) as compared to controls (4.9 ± 3.6 hours, range = 0 – 15.5 hours) (p = 0.002) (Figure 2). In multivariable logistic regression models adjusting for age, gender, race, cognition, comorbidities, and grip strength (Table 4), AMD subjects demonstrated an increased likelihood of not leaving their home on a given day (OR = 1.36, 95% CI = 1.0 – 1.8, p = 0.04). When driving status (still driving vs. not driving) was added to the multivariable model (not shown in table), the association between AMD status and excursions lessened (OR = 1.29) and the association was no longer statistically significant (p = 0.11). Greater odds of not leaving the home on a given day was also associated with worse VA (OR = 1.08 per 0.1 logMAR decrement, 95% CI = 1.0 – 1.2, p = 0.05) and older age (OR = 2.20 per 10 year increase in age, 95% CI = 1.3 – 3.7, p = 0.002). In separate multivariate models, worse CS was not associated with an increased likelihood of not leaving the home on a given day (p = 0.12).

**Table 4:** **Effect of AMD and vision metrics on the likelihood of not leaving home on a given day**

Variable	Interval	Univariate analysis		Multivariate analysis	
		OR	***P***value	OR*	***P***value
**Vision**					
AMD	Present	1.59	**<0.001**	1.36	**0.04**
Better eye VA	0.1logMAR worse	1.13	**<0.001**	1.08	0.07
Binocular CS	0.1 log decrement	1.11	**0.006**	1.06	0.12
Sig. cataract/PCO	Present	1.25	0.46	---	---
**Demographics**					
Age	10 years older	3.00	**<0.001**	2.20	**0.002**
Gender	Female vs. male	0.79	0.44	0.56	0.19
Race	White vs. non-White	1.13	0.32	1.09	0.39
Education	4 years more	1.39	0.22	---	---
Employed	Yes vs. No	0.37	**0.007**	§§	§§
**Health/cognition /driving**					
Comorbidities	1 or more illness	1.02	0.84	0.97	0.79
Depressive symptoms	Present vs. absent	1.61	0.39	§§	§§
BMI	1 unit higher	1.02	0.30	---	---
MMSE – VI score	5 points lower	2.33	**0.03**	1.40	0.35
Grip strength	5 kg less	1.07	0.25	1.21	0.06
**Weather**					
Temperature <45°F	Yes vs. No	1.07	0.66	---	---
Rainfall > 0.2 mm	Yes vs. No	0.94	0.60	---	---

OR = Odds ratio of not leaving home on a given day; AMD = age-related macular degeneration; VA = visual acuity; CS = contrast sensitivity; logMAR = logarithm of the minimum angle of resolution; Sig. = significant; PCO = Posterior Capsular Opacification (either eye); BMI = body mass index’ MMSE-VI = Mini-mental status examination for the visually impaired; °F = degrees Fahrenheit; mm = millimeters.

*OR for vision variables were each derived from separate multivariable models including all non-visual covariates shown. OR for non-visual variables were derived from the multivariable model containing AMD as the vision variable. Values in bold indicate p < 0.05.

§§ - Employment was not included in the multivariable models as unemployment may occur as a result of AMD and does not directly influence effect of vision related parameters on travel away from home.

Time away from home was considered as a final mobility outcome. In multivariate analyses adjusting for age, gender, race, cognition, comorbidities, and grip strength, fewer hours away from home were spent by subjects who were older (34% reduction per 10-year increment in age, 95% CI = −18 to −46%, p < 0.001; Table 5) and who had weaker grip strength (9% reduction per 5 kg decrement, 95% CI = −2 to −15%, p = 0.006). Women spent significantly more time away from home (44% increment compared to men, 95% CI = 1 – 93%, p = 0.04). The presence of AMD, worse VA, and worse CS were each associated with less time spent away from home in separate multivariate models, though these associations were not statistically significant (p = 0.11, 0.09, and 0.33, respectively).

**Table 5:** **Effect of AMD and AMD severity on time spent outside the home**

Variable	Interval	Univariate analysis		Multivariate analysis	
		RR	***P***value	RR*	***P***value
**Vision**					
AMD	Present	0.82	**0.001**	0.91	0.11
Better eye VA	0.1logMAR worse	0.93	**0.003**	0.97	0.09
Binocular CS	0.1 log decrement	0.95	**0.03**	0.98	0.33
Sig. cataract/PCO	Present	1.00	0.98	---	---
**Demographics**					
Age	10 years older	0.59	**<0.001**	0.66	**<0.001**
Gender	Female vs. male	1.19	0.16	1.40	**0.04**
Race	White vs. non-White	0.95	0.46	0.94	0.26
Education	4 years more	0.87	0.37	---	---
Employed	Yes vs. No	1.22	**0.02**	§§	§§
**Health/cognition /driving**					
Comorbidities	1 or more illness	0.97	0.41	1.00	0.98
Depressive symptoms	Present vs. absent	0.94	0.78	---	---
BMI	1 unit higher	0.99	0.86	---	---
MMSE – VI score	5 points higher	0.83	0.28	1.08	0.63
Grip strength	5 kg less	0.97	0.36	0.91	**0.02**
**Weather**					
Temperature <45°F	Yes vs. No	1.00	0.97	---	---
Rainfall >0.2 mm	Yes vs. No	0.99	0.83	---	---

RR = Rate ratios; AMD = age-related macular degeneration; VA = visual acuity; CS = contrast sensitivity; logMAR = logarithm of the minimum angle of resolution; PCO = Posterior Capsular Opacification; BMI = body mass index; MMSE-VI = Mini-mental status examination for the visually impaired; °F = degrees Fahrenheit; mm = millimeters.

*RR for vision variables were each derived from separate multivariate models including all non-visual covariates shown. RR for non-visual variables were derived from the multivariate model containing AMD as the vision variable. Values in bold indicate p < 0.05.

§§ - Employment was not included in the multivariable models as unemployment may occur as a result of AMD and does not directly influence the effect of vision related parameters on travel away from home.

The influence of age and race on primary outcome variables were further explored in models restricted by age and race. When multivariate analyses were restricted to white patients over the age of 70, similar rate ratios or odds were observed for the association between the degree of vision impairment and the study outcomes of steps, time spent in MVPA, time spent away from home, and excursions.

## Discussion

In this clinic-based sample of patients, those with AMD took fewer steps and spent less time engaged in physical activity when compared to those with normal vision, and a significant dose-dependent relationship was noted between worse vision and daily physical activity. The impact of AMD-related central vision impairment on daily MVPA was noted to be significantly greater than the impact of having an additional comorbid illness. Subjects with AMD were also less likely to leave the home than normally-sighted subjects. Additionally, AMD subjects spent less time away from home compared to controls, although this finding was not statistically significant. The fact that frequency of excursions was significantly associated with AMD while time spent away from home was not may be explained by the fact that some AMD subjects spend more time on their excursions, possibly as a result of slower movement [29] and/or a desire to spend more time away from home during each excursion. Our results indicate that mobility metrics which are strongly predictive of health are significantly affected in AMD and are more strongly impacted in those patients with worse vision.

Our findings add to a growing body of evidence that suggests a strong association between vision and accelerometer-defined physical activity [5–7]. Previous studies such as the National Health and Nutrition Examination Survey (NHANES) have demonstrated a strong association between worse best-corrected VA and physical activity, though the cause of decreased VA in NHANES was not determined [6]. Of note, the 35% decrement in MVPA amongst our AMD group was less than the 48% decrement in MVPA reported for NHANES subjects with visual acuity of 20/50 or worse. However, the AMD subjects in the current study had a median VA of 20/45, and 46% saw 20/40 or better. Encouragingly, we found that some patients with poor vision from AMD continued to be physically active suggesting that increasing physical activity through more steps and more vigorous activity is a realistic rehabilitation goal.

We found that a 0.1 logMAR difference in VA had a similar, though slightly lower, impact on physical activity as was noted for a 5-dB change in patients with glaucoma [7]. Furthermore, CS had an impact on activity in both AMD and glaucoma. For example, amongst glaucoma patients a 5-dB VF MD decrement was associated with 17% less time spent in MVPA, and a 0.1 decrement in logCS was associated with 6% less time spent in MVPA [7]. In this study, subjects with 0.1 logMAR worse VA spent 11% less time in MVPA, and those with 0.1 log worse CS spent 13% less time in MVPA. These data support the concept that significant physical activity limitations occurs with either central or peripheral vision impairment as well as impairment in CS, though further work is necessary to determine whether similar mechanisms underlie activity restriction in both types of vision impairment.

Our findings are also consistent with prior research demonstrating life space constriction in those with central vision impairment. Popescu et.al. compared questionnaire-derived life space scores in patients with vision loss from AMD, glaucoma, Fuchs’ endothelial dystrophy, and controls with normal vision, reporting that AMD subjects had the highest life space constriction compared to subjects with glaucoma and Fuchs’ endothelial dystrophy [8]. While this finding is consistent with those of the current study, life space questionnaires have limitations including their reliance upon self-report and the fact that clinical interpretation of their output is more difficult to assess than the direct measurement of excursions offered by tracking devices. For example, while Popescu et al. found that life space scores were roughly 16 points lower for AMD subjects as compared to controls, we were able to demonstrate in our current study that AMD patients had a 1.36 fold higher odds of not leaving home on a given day, and were likely to spend 9% less time outside the home, as compared to controls – outcomes which are more easily understood.

There is strong evidence supporting the fact that physically active older adults have improved cardiovascular, metabolic, and cognitive health [30–32]. Conversely, previous studies have suggested that failure to engage in regular physical activity or travel outside the home has a negative impact on health and well-being. One community-based longitudinal study found that the risk of mortality was 20% higher in subjects with home confinement and constricted life space [33]. Additionally, individuals with life space restriction are more socially isolated, have worse nutrition [34], are more frail [3], and have a greater rate of incident mortality [3, 35], Alzheimer disease [36] and cognitive decline. These previous data, combined with our current data, suggest that interventions to encourage physical activity and travel outside the home might improve health, well-being, and longevity when targeted to older adults with vision loss from AMD.

Identifying intermediaries in the pathway between vision loss and mobility is critical for developing interventions to avoid mobility restriction. When driving status and VA were added to the multivariable model analyzing the odds of leaving one’s home, attenuation as well as a loss of statistical significance was present between AMD status and frequency of excursions, suggesting that VA and driving (which may be a marker of VA) may at least partially mediate the impact of poorer vision on the likelihood of leaving one’s home. This finding highlights the importance of alternative means of transportation to leave the home. Other potential mediators between vision and mobility restriction, such as fear of falling, should be studied in order to identify modifiable risk factors for decreased physical activity and travel outside the home.

The results from the current study may not be generalizable to all AMD patients, as subjects in our study were selected from patients who visit an urban tertiary care center, which potentially excluded individuals with the greatest physical activity or life space restrictions. Furthermore, individuals with more restricted mobility may have been less inclined to participate in this study due to the need for additional study visits, thus resulting in potential underestimation of the impact of AMD on mobility. This limitation was mitigated by offering for participants to complete their testing on the same day as their clinical visits. Finally, while the technology used to evaluate mobility provided reliable and quantifiable measurements, a limitation of accelerometers is that they do not accurately calculate the calories burned during activities such as cycling and swimming. Despite this limitation, there is a large body of literature showing that accelerometers do capture true physical activity levels significantly better than questionnaires [10].

## Conclusion

In conclusion, individuals with AMD do not engage in physical activity nor leave their homes as much as normally-sighted individuals. In view of the many psychological and health benefits of being physically active and spending time outside the home, it is important to find innovative ways to promote safe physical activity and travel away from home. Determining potential factors which serve as intermediaries between vision loss of decreased mobility will offer the potential to improve mobility and thus improve quality of life and overall health in individuals with AMD.

## Authors’ information

Sabyasachi Sengupta: Aravind Eye Hospital, Thavalakuppam, Pondicherry, India 605007; Email: drsunny1980@gmail.com

Angeline M. Nguyen: The Wilmer Eye Institute, 600 North Wolfe Street, Maumenee B-110, Baltimore, MD 21287; Email: anguye24@jhmi.edu

Suzanne W. van Landingham: The Wilmer Eye Institute, 600 North Wolfe Street, Maumenee B-110, Baltimore, MD 21287; Email: swestbr3@jhmi.edu

Sharon D. Solomon: The Wilmer Eye Institute, 600 North Wolfe Street, Maumenee M-740, Baltimore, MD 21287; Email: ssolomon1@jhmi.edu

Diana v. Do: 985540 Nebraska Medical Center, Omaha, NE 68198–5540; Email: diana.do@unmc.edu

Luigi Ferrucci: Harbor Hospital, 3001 Hanover Street, Baltimore, MD 21225; Email: ferruccilu@mail.nih.gov

David S. Friedman: The Dana Center for Preventive Ophthalmology, Wilmer 120, 600 North Wolfe Street, Baltimore, MD 21287; Email: david.friedman@jhu.edu

Pradeep Y. Ramulu: The Wilmer Eye Institute, 600 N Wolfe St, Maumenee B-110, Baltimore, MD 21287; Email: pramulu@jhmi.edu

## Abbreviations

BMI: Body-mass index
MVPA: Moderate-to-vigorous physical activity
VF: Visual field
CS: Contrast sensitivity
AMD: Age-related macular degeneration
VA: Visual acuity
dB: Decibels
MD: Mean deviation
SITA: Swedish interactive thresholding algorithm
GHT: Glaucoma hemifield test
SEEDS: Salisbury eye evaluation driving study
EDTRS: Early treatment of diabetic retinopathy study
logMAR: Logarithm of the minimum angle of resolution
logCS: Contrast sensitivity converted to a log scale
PCO: Posterior capsular opacification
BMI: Body mass index
MMSE: Mini mental state exam
GEE: Generalized estimating equation
RR: Rate ratio
IQR: Interquartile range.
